# An Integrative Transcriptome Subtraction Strategy to Identify Human lncRNAs That Specifically Play a Role in Activation of Human Hepatic Stellate Cells

**DOI:** 10.3390/ncrna10030034

**Published:** 2024-06-06

**Authors:** Yonghe Ma, Jamie Harris, Ping Li, Chengfei Jiang, Hang Sun, Haiming Cao

**Affiliations:** Cardiovascular Branch, National Heart, Lung and Blood Institute, National Institutes of Health, Bethesda, MD 20892, USA

**Keywords:** long non-coding RNAs (lncRNAs), liver fibrosis, hepatic stellate cells, CARMN

## Abstract

Fibrotic liver features excessive deposition of extracellular matrix (ECM), primarily produced from “activated” hepatic stellate cells (HSCs). While targeting human HSCs (hHSCs) in fibrosis therapeutics shows promise, the overall understanding of hHSC activation remains limited, in part because it is very challenging to define the role of human long non-coding RNAs (lncRNAs) in hHSC activation. To address this challenge, we identified another cell type that acts via a diverse gene network to promote fibrogenesis. Then, we identified the lncRNAs that were differentially regulated in activated hHSCs and the other profibrotic cell. Next, we conducted concurrent analysis to identify those lncRNAs that were specifically involved in fibrogenesis. We tested and confirmed that transdifferentiation of vascular smooth muscle cells (VSMCs) represents such a process. By overlapping TGFβ-regulated lncRNAs in multiple sets of hHSCs and VSMCs, we identified a highly selected list of lncRNA candidates that could specifically play a role in hHSC activation. We experimentally characterized one human lncRNA, named CARMN, which was significantly regulated by TGFβ in all conditions above. CARMN knockdown significantly reduced the expression levels of a panel of marker genes for hHSC activation, as well as the levels of ECM deposition and hHSC migration. Conversely, gain of function of CARMN using CRISPR activation (CRISPR-a) yielded the completely opposite effects. Taken together, our work addresses a bottleneck in identifying human lncRNAs that specifically play a role in hHSC activation and provides a framework to effectively select human lncRNAs with significant pathophysiological role.

## 1. Introduction

In the past two decades, non-alcoholic fatty liver disease (NAFLD) has become a major public health threat in the US and worldwide, affecting 30% of the general population [1,2]. NAFLD is an umbrella term encompassing a spectrum of related clinical entities from simple steatosis (pure NAFLD) to non-alcoholic steatohepatitis (NASH), with possible evolution to cirrhosis and hepatocellular carcinoma [3,4]. However, the options for the therapeutic treatment of NASH-related disorders are still very limited [3]. The disease-driving pathways of NASH converge on fibrosis or its most severe and extensive pattern, cirrhosis [5,6]. Fibrosis features an aberrant wound-healing process leading to degeneration of hepatocytes and deposition of extracellular matrix (ECM), which is more resistant to treatment compared to the earlier stages [3,4,7]. The current pharmacological therapeutic options reversing fibrosis focus mainly on modulating hepatic stellate cells (HSCs), the primary ECM-producing mesenchymal cells [5]. HSCs, initially named Ito cells, are localized in the subendothelial space of Disse, representing about 10% of all resident liver cells [8,9,10]. During chronic liver injury, profibrotic mediators stimulate quiescent HSCs to transform into proliferative, migratory and fibrogenic myofibroblasts, a process that is regulated by paracrine and autocrine loops of growth factors, with transforming growth factor β (TGFβ) being the central one [11,12]. Overactivated HSCs deposit unnecessary ECM, overriding wound healing and liver regeneration, further leading to fibrosis [13]. As reversing the activated HSCs is pivotal to resolving extracellular fibrosis, the pathways regulating the fate conversion of HSCs represent attractive therapeutic targets for treating and preventing hepatic fibrosis associated with NAFLD/NASH [14].

While the importance of hHSC fate conversion in liver fibrosis is widely acknowledged, the underlying molecular mechanisms remain unclear. This knowledge gap is partly rooted in our incomplete understanding of the human genome, particularly in the regions encoding long non-coding RNA (lncRNA) genes. LncRNAs are transcripts that are at least 200 nt long and have no predicted coding potential [15]. Currently, over 60,000 lncRNA genes have been identified [16,17], and lncRNAs have been shown to regulate diverse processes in biology and physiology [18,19,20]. Due to the non-coding nature of lncRNAs, however, it remains challenging to identify human lncRNAs that play a specific pathophysiological role. Currently, the most widely used method to identify functional lncRNAs is to identify lncRNAs that are differentially regulated during a specific biological/physiological process [21,22]. For example, lncRNAs that are strongly regulated during the development of liver fibrosis could play a role in this process. However, it is known that any specific pathophysiological process often regulates the expression of hundreds and even thousands of genes; thus, further conditions are needed to narrow down those that specifically function in the process. For protein-coding genes, multiple approaches can be used to achieve this goal. For example, functional domains and motifs on a protein can be used to infer its function. Additionally, the function of a protein’s ortholog in another species can also provide important information. None of these approaches, however, are available for lncRNAs. Due to the limited understanding of the sequence–function relationship, it is currently impossible to use motifs or functional domains to place a lncRNA in a biological context. Furthermore, lncRNAs are much less conserved than protein-coding genes, and most human lncRNAs are human- or primate-specific [20,23,24]. Functional information on a human lncRNA’s homolog in other species is thus often unavailable. Further adding to the challenge, it is currently unknown what fraction of the vast number of lncRNAs are functional. Thus, effective strategies to prioritize the functional potential of human lncRNAs are particularly critical to understanding their function but are paradoxically lacking.

To understand the role of functional lncRNAs in stellate cell activation, given the challenges of selecting functional lncRNAs, we established a strategy to select human lncRNAs that specifically play a role in this process. We conceptualized that profibrogenic human lncRNAs in HSCs could be specifically identified by concurrent analysis of regulated lncRNAs during HSC activation with those in a related process under which diverse gene networks are regulated to promote fibrogenesis. We determined that TGFβ-induced transdifferentiation of vascular smooth muscle cell (VSMC) represents such a process, and a transcriptome subtraction between the two processes led to the identification of cardiac mesoderm enhancer-associated non-coding RNA (CARMN) as a candidate lncRNA that could function in hHSC activation. We further utilized CARMN gain- and loss-of-function assays to confirm its functional importance in the fibrogenesis of HSCs. Our study thus defines a lncRNA-mediated mechanism of hHSC activation and also establishes a framework to identify human lncRNAs that specifically play a role in important pathophysiological processes.

## 2. Results

### 2.1. Identifying an In Vitro Culture System That Is Suitable to Study hHSC Activation

In this work, we aimed to identify human lncRNAs that specifically play a role in hHSC activation. As human lncRNAs are challenging to study in vivo, we first sought to identify an optimal in vitro/ex vivo system to study the activation of hHSC. This is not a trivial issue, as hHSCs rapidly switch to the activated state when cultured in plastic [25], which represents an obstacle to studying HSC physiology in vitro. Many studies focus on maintaining hHSCs in quiescent state by modulating the culture conditions, showing some potential in culturing hHSCs using 3D systems [8,26,27,28]. Therefore, we performed an extensive survey of current hHSC 3D and 2D culture systems to identify a system that is most suitable to study HSC activation. The three 3D systems tested were culture on a low attachment plate with constant shaking, culture in high-viscosity media and culture in Elplasia Plates. To evaluate the feasibility of 3D culture in initiating hHSC activation, we stained cultured spheroids for pan-collagen and α-SMA, two markers of fibrogenesis. We found that both were significantly induced by TGFβ (Figure S1). Additionally, we cultured P1 human primary HSC from two donors, as well as LX-2, a routinely used immortalized hHSC cell line featuring key characters of pHSC.

**Figure 1:**
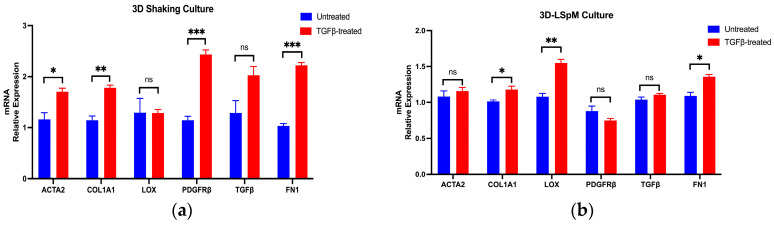

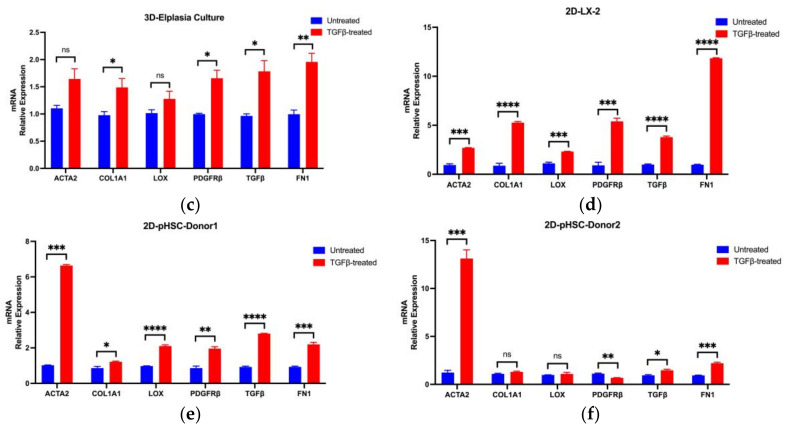
TGFβ induced expression of HSC activation markers in different 2D or 3D culture conditions. (**a**) 3D culture of pHSC on a low attachment plate with constant shaking; (**b**) 3D culture of pHSC in high-viscosity media; (**c**) 3D culture of pHSC in Elplasia plate; (**d**) 2D culture of LX-2 cell© (**e**) 2D culture of pHSC from Donor 1; (**f**) 2D culture of pHSC from Donor 2. Cells were treated with 5 ng/mL of TGFβ for 24 h before collection for RNA isolation and RT-qPCR. For comparisons between two groups, a two-tailed, unpaired Student’s *t*-test was used. A *p*-value of more than 0.05 was considered non-significant (ns); a *p*-value between 0.01 and 0.05 was considered significant (*); a *p*-value between 0.001 and 0.01 was considered very significant (**); a *p*-value between 0.0001 and 0.001 was considered very extremely significant (***); a *p*-value of less than 0.0001 was considered extremely significant (****).

We generated a panel of markers to evaluate the extent of hHSC fate conversion to the myofibroblast-like phenotype in these systems. When converting from HSCs, myofibroblasts share two standard features: de novo expression of smooth muscle alpha-actin (α-SMA) and abundant ECM, mainly type I collagen [29]. We thus chose actin alpha 2, smooth muscle (ACTA2) and collagen type I alpha 1 chain (COL1A1), as they play an essential role in many cell processes, including cell proliferation, differentiation, migration and contraction [30]. Lysyl oxidase (LOX) activity contributes significantly to collagen stabilization [31], while fibronectin (FN1) induces cell cycle progression [32]. We also included two secreted factors: TGF-β1 and PDGFRβ. TGF-β1 can be secreted by activated HSCs and further contribute to the activation of hHSCs, promoting the fibrosis progression [29]. Platelet-derived growth factor receptor beta (PDGFRβ) is a cell surface tyrosine kinase receptor, which is augmented in active HSC and functions in enhancing the responsiveness of the HSCs toward various stimulating factors secreted by their microenvironment [33]. As shown in Figure 1, the relative mRNA expression levels of the activation markers were varied after TGFβ treatment in the different culture conditions. Although most of the markers were induced in 3D systems, the extent of the induction did not appear to be superior to those in 2D systems. We also found that the overall patterns of gene induction in LX-2 cells were the most robust, and the overall patterns closely matched those in hHSCs. Thus, we decided to use LX-2 to select to functionally test human lncRNAs that could play a role in hHSC activation.

### 2.2. Identification of VSMC Transdifferentiation as a Functionally Relevant System That Shares the Key Elements of Fibrogenesis with hHSC Activation

The most common approach to identify genes that specifically function in a physiological process is to identify and test those genes that are differentially regulated by the process. However, the study of lncRNAs provides further complications, as the regulated pool is often enormous, with limited effective downstream selection approaches. To address this challenge, we sought to conduct a hypothesis-driven transcriptome subtraction strategy. We first identified another cell type that acts via a diverse gene network to promote fibrogenesis. Then, we identified the lncRNAs that were differentially regulated in activated hHSCs and the other profibrotic cell to conduct concurrent analysis and identify those lncRNAs that were specifically involved in fibrogenesis. It is known that activated HSCs up-regulate genes related to cell proliferation and migration [34]. They also immediately attach and switch into a myofibroblast-like phenotype when cultured on plastic, displaying well-developed stress fibers of the actin cytoskeleton and increasing proliferation [35]. This process not only resembles what is observed in chronic fatty liver diseases but also shows remarkable similarities to the transdifferentiation of vascular smooth muscle cells (VSMCs). In response to vessel injuries, VSMCs switch from a contractile to a synthetic phenotype while acquiring new cellular functions, contributing to vascular wall remodeling, including migration and proliferation. Moreover, arterial VSMCs become aberrantly regulated in atherosclerosis, leading to excess VSMC dedifferentiation and extracellular matrix formation in plaque areas [36,37,38]. VSMC phenotype conversion is a complex and multistep mechanism that can be induced by various stimuli, including TGFβ [39,40]. We hypothesized that HSCs and VSMCs may share the key elements of fibrogenesis, as they share physiologically relevant similarities in their cell lineage, function and in vitro behavior. As two terminally differentiated cell types, HSCs and VSMCs share the same neural crest lineage in development, though there is still some debate [41]. In anatomy, HSCs are closely associated with the sinusoidal endothelial cells and make contact to the endothelium in an analogous way as VSMCs to capillaries [10]. In vitro activated HSCs behave very similar to VSMCs, as HSCs can replace VSMCs in a functional three-dimensional spheroidal co-culture system relating angiogenesis [42].

**Figure 2:**
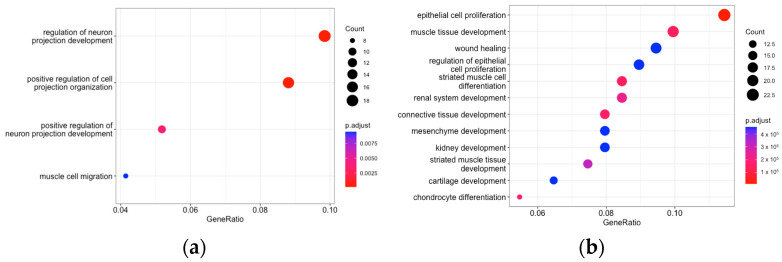
Dot plot showing GO pathway analysis of differentially expressed coding genes overlapped between HSC and macrophages (**a**) or HASMC (**b**) upon TGFβ treatment.

To test our hypothesis that HSCs and VSMCs share the key elements of fibrogenesis, we compared the TGFβ-induced changes in gene expression regulation in hHSCs to those in four different cell types: endothelial cells, smooth muscle cells, CD4+ effector memory T cells and activated macrophages. We first analyzed a published dataset (GSE134555) to identify differentially expressed genes (DEGs) in the four cell types that were treated with TGFβ. We then overlapped the DEGs from each cell type with those in TGFβ-treated hHSCs. The overlapped protein-coding genes were then subjected to Gene Ontology (GO) pathway analysis. For endothelial cells and CD4+ effector memory T cells, only a small number of genes, i.e., 44 and 27, overlapped with those in TGFβ-treated hHSCs (Table S1), suggesting that their overall responses to TGFβ are very different from hHSCs. In contrast, 208 and 210 TGFβ-regulated genes in smooth muscle cells and macrophages, respectively, overlapped with those in hHSCs (Table S1). The GO analysis indicated that TGFβ-regulated genes in macrophages were limited to a few pathways, and none of them were related to the core features of hHSC activation (Figure 2a). In contrast, multiple top pathways from smooth muscle cells were related to proliferation and wound healing, and this result confirmed our hypothesis that smooth muscle cells and hHSCs share the molecular elements of fibrogenesis (Figure 2b).

### 2.3. Bioinformatic Screening of lncRNAs Functioning in HSC/VSMC Transdifferentiation

**Figure 3:**
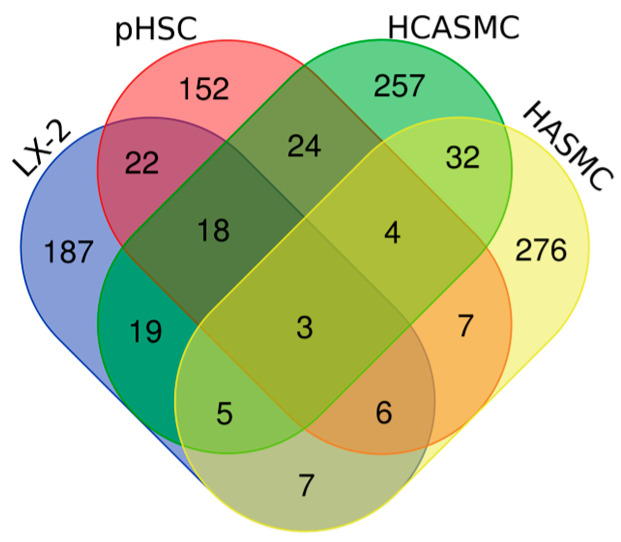
Venn diagram showing the numbers of overlapped lncRNAs that were regulated in LX-2, pHSC, HCASMC and HASMC by TGFβ treatment.

After confirming that a set of fibrogenic genes were similarly induced in hHSCs and VSMCs by TGFβ, we decided to perform a transcriptome subtraction of regulated lncRNAs between the two conditions to identify lncRNAs that could specifically play a role in HSC activation. In total, we overlapped DEGs from five datasets. The first two datasets reflected HSC fate conversion. We performed RNA-Seq analyses of LX-2 cells and hHSC from one representative donor (pHSC, Donor 1) under control or TGFβ-treated conditions. The following two datasets downloaded from Gene Expression Omnibus (GEO) reflected VSMC transdifferentiation under TGFβ treatment, which were control or TGFβ-induced human coronary artery smooth muscle cell (HCASMC, GEO, GSE85910) and human aortic smooth muscle cell (HASMC, GEO, GSE134555), respectively. We used a stringent statistical cut-off (logFC > 1, *p* < 0.01) to select lncRNAs that exhibited significant TGFβ-induced expression changes in all datasets (Figure 3), which led to three lncRNAs: linc01711, CARMN and RP11-154H23.3. Although the data above provide valuable information for distinct tissue-relevant lncRNAs, they only reflect the regulated lncRNAs in a restricted donor pool, as some of the cells described above, such as pHSC, were derived from a single donor. Thus, it is unknown whether the selected lncRNAs are applicable to the general population. To evaluate this, we analyzed an RNA-Seq dataset of primary VSMCs from 22 patients (GSE142417) with diverse genetic backgrounds and found that all three lncRNAs were regulated by TGFβ in these cells. This result supports the notion that lncRNA regulation by TGFβ is not dependent on a specific genetic background and could be extrapolated to the general population.

Although the same lncRNAs may exhibit overlapping expressions in different cell types, they could still serve different functions. To predict the function of these three screened lncRNAs above, we performed a correlation analysis of identified lncRNAs using gene expression data in VSMC and stellate cells to infer their function (Table S4). We found that the top pathways that correlate with CARMN in the stellate cell are related to TGFβ and extracellular matrix, which is consistent with its function in stellate cell activation (Figure S3a). Interestingly, the pathways in the VSMC database using the same method are centered on smooth muscle contraction, which also aligns well with CARMN’s known function (Figure S3b). For the other two lncRNAs, the top pathways enriched in stellate cells resemble those shown in CARMN. This further supports our screen pipeline’s reliability (Figure S3c,e). Although some relevant pathways pop up from the result in VSMC for linc01711 and RP11-154H23.3, they are relatively less represented, which implies that these two lncRNAs may have other VSMC-specific functions (Figure S3d,f). Among all three lncRNAs, RP11-154H23.3 is poorly annotated and studied. Linc01711 is an intergenic lncRNA, which has a relatively clear genetic structure. We found at least two studies demonstrating its role in fibrosis [43,44]. CARMN was first identified and characterized as a super-enhancer-associated lncRNA regulating cardiac specification, differentiation and homeostasis [45]. Several reports have detailed the function of CARMN in VSMC [46,47], but no study of its function in HSCs has been published. Taking all the rationales into consideration, we selected CARMN for further research.

### 2.4. CARMN Expression in Stellate Cells Is Induced by TGFβ

**Figure 4:**
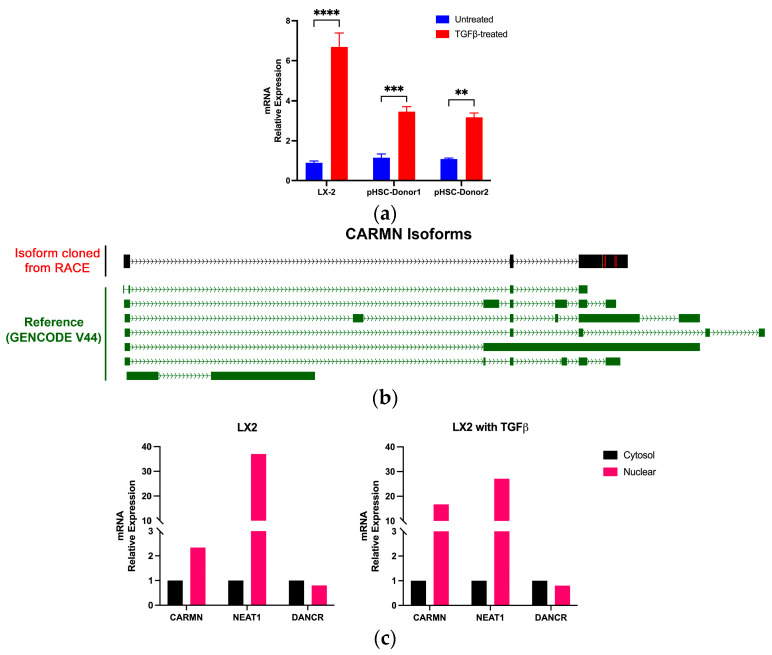
Characterization of stellate-cell-expressed CARMN. (**a**) CARMN expression is induced by TGFβ treatment in LX-2 and two donors of hHSCs; (**b**) Exons’ schematic comparison between newly cloned CARMN transcript from RACE and reference from GENCODE V44. The newly cloned CARMN isoform with three exons is uniquely expressed in LX-2 cells. The red strip shows where the genome and cloned sequence have different bases at this position, while the orange shows an insertion (or the genome has a deletion/alignment gap) at this point; (**c**) Cellular fractionation of CARMN in LX-2 with (left) or without TGFβ treatment (right). The cytosolic expression is set as 1. The nuclear fraction is depicted as fold changes relative to the expression in the cytosol. For comparisons between two groups, a two-tailed, unpaired Student’s *t*-test was used. A *p*-value between 0.001 and 0.01 was considered very significant (**); a *p*-value between 0.0001 and 0.001 was considered very extremely significant (***); a *p*-value of less than 0.0001 was considered extremely significant (****).

To validate the regulation of CARMN in HSC by TGFβ, we performed quantitative reverse transcription PCR (qRT-PCR) analysis of RNAs isolated from LX-2 cell lines and hHSCs derived from two donors (Figure 4a). We confirmed that CARMN was expressed in all tested cell lines and was significantly up-regulated when the cells were treated with TGFβ, which further supports the assumption that CARMN may be involved in HSC state conversion.

LncRNAs often have tissue-specific transcripts or isoforms, which is closely related to tissue-specific functions. Multiple isoforms of CARMN have been reported in common databases, but only the 5′ ends of these annotated transcripts are relatively consistent. To further characterize hHSC-expressed CARMN, we performed rapid amplification of cDNA ends (RACE) to characterize the 3′ end of the full-length CARMN expressed in the LX-2 cell line. This analysis identified a novel 3′ end of CARMN, which is unique and has not been annotated. Using the annotated 5′ end and the new 3′ end information, we amplified the full-length CARMN in HSC, which shows a similar structure to previously published CARMN isoforms but with a unique third exon on the 3′ end (Figure 4b). As alternative splicing can dictate the function of a certain gene in various cell types, the HSC-expressed CARMN may serve unique functions specific to the HSC activation process.

To determine the cellular distribution of CARMN, we conducted a cell fractionation assay in TGFβ-treated and control cells in LX-2 and found CARMN to be localized in the nucleus of LX-2 cells, particularly when cells were treated with TGFβ (Figure 4c). CARMN was present in a greater proportion in the nuclear fraction. We used NEAT1 and DANCR, well-studied nuclear and cytosolic lncRNAs, respectively. The location of CARMN most resembles that of the nuclear lncRNA, NEAT1, and is further expressed during TGFβ induction.

### 2.5. Loss of Function of CARMN Attenuates HSC Activation

**Figure 5:**
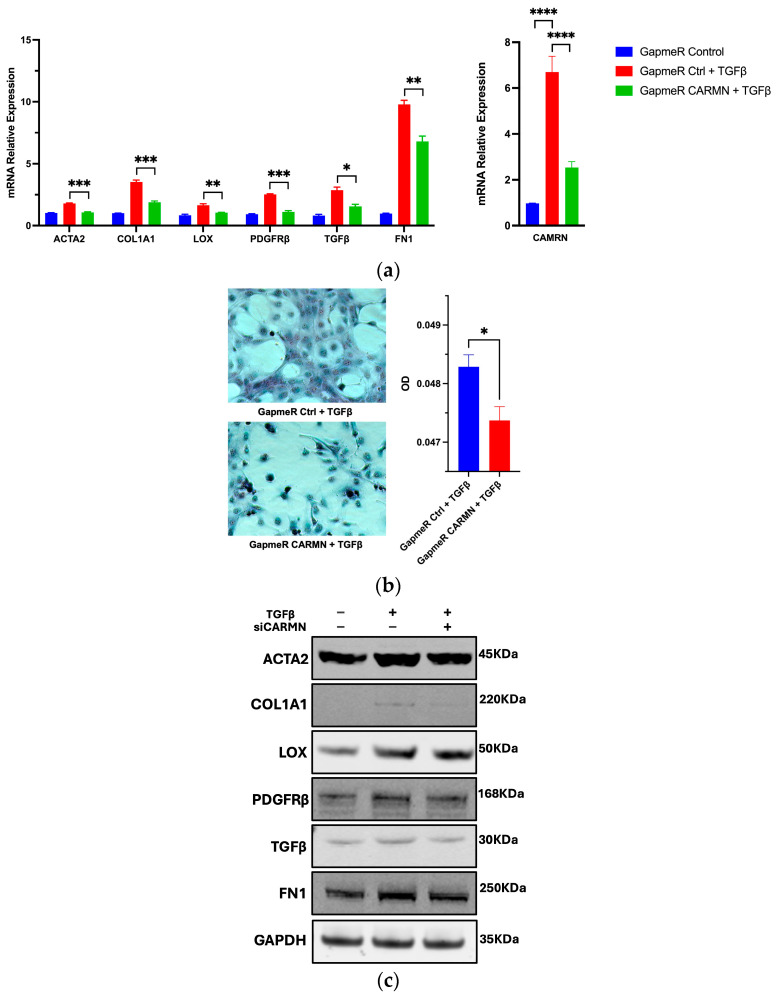
CARMN loss of function attenuates HSC activation. (**a**) Expression of HSC activation panel makers using anti-CARMN GapmeR to knock down CARMN with TGFβ treatment (*n* = 3). The knockdown efficiency is shown side by side. The result is representative of three repeated experiments; (**b**) Western blot analysis of marker gene changes with CARMN knockdown. Molecular weights are labeled on the right. The images shown are the representative images of three repeated experiments. The GAPDHs were loaded as the control for blotting COL1A1; (**c**) Collagen staining and signal quantification (*n* = 6) showing the expression change in total collagen amount change with CARMN knockdown under TGFβ treatment. Data are shown as the mean ± SEM. Statistical analyses between two groups are conducted using two-tailed, unpaired Student’s *t*-test. A *p*-value between 0.01 and 0.05 was considered significant (*); a *p*-value between 0.001 and 0.01 was considered very significant (**); a *p*-value between 0.0001 and 0.001 was considered very extremely significant (***); a *p*-value of less than 0.0001 was considered extremely significant (****).

After confirming that CARMN is dynamically regulated in stellate cells, we examined whether loss of function of CARMN has an impact on stellate cell activation phenotypically. We knocked down CARMN using Antisense LNA GapmeR and assessed the expression of a panel of stellate cell activation markers. Interestingly, CARMN knockdown in LX-2 cells resulted in a significantly decreased expression of stellate cell activation markers induced by TGFβ (Figure 5a). To circumvent the off-target effect of the GapmeR tool, we used two anti-sense oligos (ASO-1 and ASO-2) targeting CARMN to knock down CARMN under the same condition and obtained a similar result (Figure S4). We then evaluated the protein level change in the markers by Western blot and found that their protein expressions were reduced in the setting of concurrent TGFβ treatment and knockdown of CARMN, which was consistent with the mRNA level change (Figure 5b). The collagen staining result comparing the control and CARMN knockdown conditions under TGFβ treatment further reinforced the phenotypic change induced by CARMN knockdown via both qualitative and quantitative analysis of collagen staining (Figure 5c).

**Figure 6:**
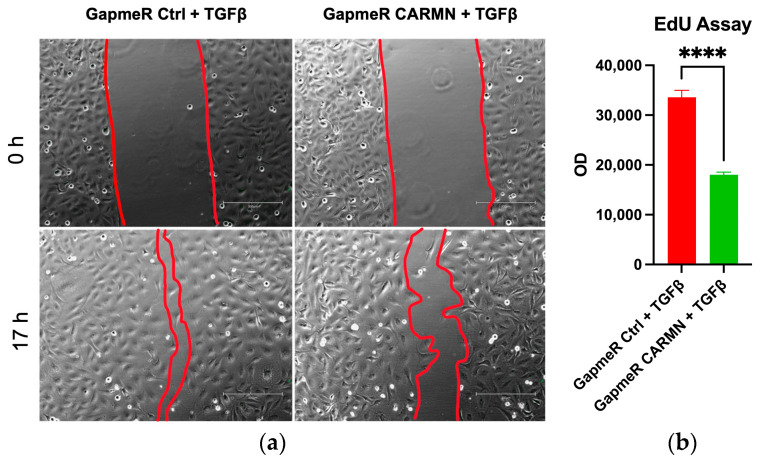
CARMN loss of function leads to decreased cellular migration and proliferation under TGFβ treatment. (**a**) Wound healing assay of LX-2 cells treated under TGFβ treatment between control and CARMN knockdown group. The scratch was made at 0 h, and the space showing in red lines was delineated after 17 h. The result is representative of three repeated experiments; (**b**) EdU assay to evaluate cell proliferation rate. Data are shown as the mean ± SEM. Statistical analyses between two groups are conducted using two-tailed, unpaired Student’s *t*-test. A *p*-value of less than 0.0001 was considered extremely significant (****).

Next, we conducted a wound healing assay to examine whether these changes resulted in migration-related phenotypic changes. This assay was used to evaluate the wound healing ability of stellate cells, independent of cell proliferation, as the cells were treated with a chemotherapeutic agent, mitomycin C, to block cell proliferation. We found that LX-2 cells under CARMN knockdown treatment healed more slowly than the control counterpart (Figure 6a). This suggests that the expression of CARMN has a phenotypically significant role in the activity of stellate cells in cell migration under wound healing conditions. Furthermore, we performed the EdU incorporation assay, which evaluates cell proliferation by measuring the rate of EdU incorporated into cellular DNA during replication [48]. In cells with CARMN knockdown, there was a significant decrease in incorporation under the TGFβ treatment condition (Figure 6b). This suggested that CARMN could also modulate HSC proliferation. Collectively, the decrease in cell proliferation, collagen expression and wound healing abilities after CARMN knockdown supports the notion that it has profibrogenic effects in hHSCs.

### 2.6. Gain of Function of CARMN Augments HSC Activation

**Figure 7:**
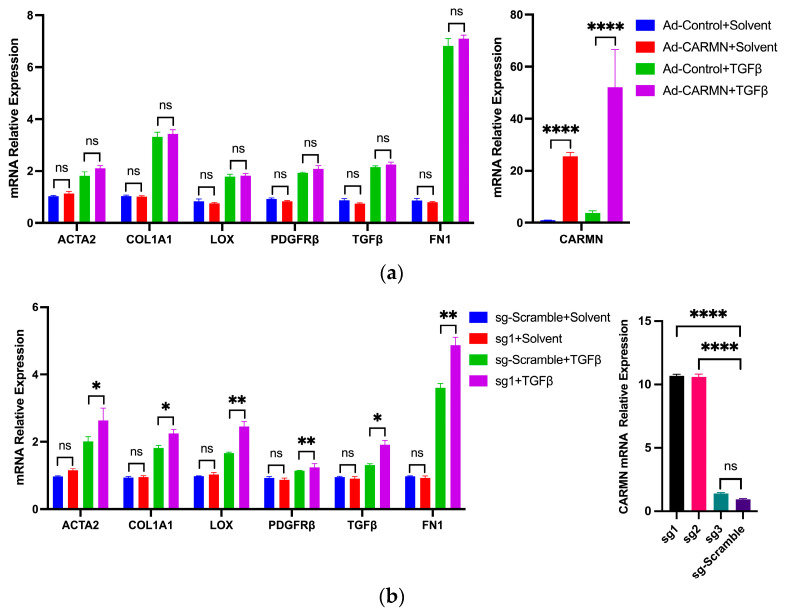
CARMN gain of function augments HSC activation. (**a**) Overexpression of CARMN using adenovirus in LX-2 cells does not further increase the expression of HSC activation marker genes induced by TGFβ (*n* = 3). The overexpression fold change is shown on the right; (**b**) Overexpression of CARMN using CRISPR activation in LX-2 cells further increases the expression of HSC activation marker genes induced by TGFβ (*n* = 3). Three sgRNAs were designed for testing, and the induction efficiency of CARMN relative to scramble sgRNA is shown on the left. For comparisons between two groups, a two-tailed, unpaired Student’s *t*-test was used. A *p*-value of more than 0.05 was considered non-significant (ns); a *p*-value between 0.01 and 0.05 was considered significant (*); a *p*-value between 0.001 and 0.01 was considered very significant (**); a *p*-value of less than 0.0001 was considered extremely significant (****).

As loss of function of CARMN down-regulated the genes involved in HSC activation, we next examined whether gain of function of CARMN had the opposite effect. First, we generated CARMN overexpression adenoviruses and used them to infect LX-2 cells. This treatment led to a dramatic induction of CARMN expression; however, the enhanced CARMN expression did not further increase the expression of marker genes under the TGFβ treatment condition (Figure 7a). To exclude the possibility that supra-physiologic CARMN expression led to these results, we adjusted CARMN’s expression by using adenovirus titer to reduce the CARMN fold change to be more physiologically relevant; yet, a similar unchanged pattern was obtained It is known that some lncRNAs use the 3D structure chromosome to bind to proteins and transport to specific locations to carry out their function [49]. Thus, the artificially overexpressed CARMN may not be able to transport to the right location required for its proper function. To this end, we used a CRISPR activation (CRISPR-a) mediated approach to specifically enhance the expression of endogenous CARMN. We screened three sgRNAs, and sg1 and sg2 showed significant CARMN induction compared with sg-Scramble (Figure 7b). When sg1 or sg2 were combined to increase CARMN expression, TGFβ-treated LX-2 cells experienced further induction of the expression of HSC activation marker genes (Figure 7b). Consistent with the effects of CARMN loss of function, this result supports the critical role of CARMN in HSC activation.

## 3. Discussion

In this study, we established a novel strategy to identify human lncRNAs that specifically play a role in hHSC activation. As hHSC is difficult to study in vivo, we first identified an in vitro cell culture system that is suitable to study hHSC activation. We then performed a hypothesis-based transcriptome subtraction to identify a small number of human lncRNAs that have functional potential in hHSC activation. We used gain- and loss-of-function approaches to confirm that one of the selected lncRNAs, CARMN, indeed regulated hHSC activation in our model.

Our selection strategy encompassed three components. First, we developed a hypothesis-based approach to identify a physiologic condition that shares the key fibrogenic elements with hHSC activation and then used transcriptome subtraction to select human lncRNAs that function in both processes. As the differential regulation of lncRNAs is often the only information available to connect lncRNAs to their specific function, our approach using two related physiologic processes to filter the differentially regulated lncRNAs could substantially increase the specificity of the selection. In biology and physiology, there are a large number of processes that are related but only share specific molecular elements. Our approach can be adapted to identify human lncRNAs that play a role in a wide range of physiological or disease conditions. Furthermore, as this approach relies on gene expression regulation, the huge repertoire of RNA-Seq data deposited in public databases such as GEO provide a nearly unlimited data source to back-test any hypothesis before carrying out experimental validation. Second, we used at least two datasets generated under each condition to subtract the transcriptomes to narrow down the specific lncRNAs. Overlapping multiple RNA-Seq datasets of diverse conditions is aimed to eliminate the noise associated with each condition. As downstream selection approaches for lncRNAs are limited, this step would help reduce non-specific regulation that is associated with each experimental condition. Third, we used datasets representing broader genomic variation in the human population to select population-applicable lncRNAs. The regulation of gene expression in cells or tissues derived from a single donor could be strongly influenced by the individual’s genetic background. To ensure that any selected lncRNAs are relevant to the general population, we further used a dataset of individuals with diverse genetic backgrounds to filter the candidate list. In the end, although this overly stringent approach often results in a short list of candidate lncRNAs, many of them are expected to carry out the specific function that they are selected for, as is the case for CARMN.

The identification of CARMN as a human lncRNA that regulates fibrogenesis could provide valuable insights into hHSC activation. Our loss- and gain-of-function studies showed that CARMN is required for TGFβ to induce the expression of fibrogenic genes and cell migration, suggesting that blocking the action of CARMN could potentially ameliorate hHSC activation in fibrotic liver. Interestingly, the overexpression of CARMN by an adenoviral tool does not induce any phenotypical change in stellate cell activation, while CRISPR-based overexpression of CARMN results in a significant augmentation of relevant genes. As CRISPR-activation-induced CARMN expression occurs locally, CARMN may function as a cis-acting lncRNA, which relies on the chromosome structure to bind to proteins and transport them to specific locations to carry out their function. It is worth motioning that although three designed sgRNAs are located in the prompter region of CARMN and, theoretically, should not have any differences except the sequences themselves, the real efficiency of these sgRNAs can only be verified by experiments. In CRISPR-based gene activation, it is common that some designed sgRNAs do not work as expected, and there are multiple potential reasons for this. For example, there could be non-specific binding of the sgRNA to other loci of the genome, which significantly dilutes its targeting effects on CARMN. Another possibility could be the presence of special chromatin/epigenetic modifications, rendering the sgRNAs inaccessible to that locus. It is currently unknown how CARMN exerts these effects, but the unique approach with which CARMN was identified could provide some interesting leads. In the two reports that characterized CARMN in VSMCs, it was shown that CARMN regulates VSMC plasticity via Myocardin- or SRF-dependent signaling pathway, respectively [46,47]. Interestingly, both pathways also regulate the expression of ACTA2, which is also a downstream target gene of CARMN in hHSCs, as shown in our study. We are currently exploring whether these pathways identified in VSMCs operate in hHSCs and whether they are the underlying mechanism through which CARMN modulates hHSC activation. Future work is centered on using more complex and relevant models to validate whether CARMN is involved in the pathogenesis of fibrosis, as our current in vitro LX-2 cell model is singular, which may not fully recapitulate the full spectrum of liver fibrosis in vivo.

In summary, our work presents a novel strategy to identify human lncRNAs that play a specific role in hHSC activation. We confirmed that one of the selected lncRNAs, CARMN, indeed regulated the key aspects of hHSC activation. Furthermore, our workflow can be adapted to investigate any physiological processes. We hope that our approach can streamline the identification of human lncRNAs that are crucial players in physiology and disease.

## 4. Materials and Methods

### 4.1. Culture and Treatment of Human HSC

Human primary HSCs (pHSCs) were purchased from Cryo Human Stellate Cells (Lonza, Walkersville, MD, USA). The donor information is shown in Table S3. Cells were thawed in Human Stellate Cell Growth Media (Lonza, Walkersville, MD, USA). For the 2D culture, pHSCs were seeded at a density of 4000–5000 cells/cm^2^ on a Collagen I coated plate using the growth media above. The media were changed every other day. Cells from the 3D culture were also purchased from Cryo Human Stellate Cells (Lonza, Walkersville, MD, USA). For pHSC cultured on a low attachment plate, 5000 cells were seeded in one well of the Ultra-Low Attachment Culture Plate (Corning, Corning, NY, USA) suspended in the growth media with constant shaking at 800 rpm. The media were carefully changed every other day without dislodging the spheroids. For cells cultured in Elplasia plates, thawed cells were resuspended in the growth media, with 200 cells seeded per microcavity in Elplasia Round Bottom Plates (Corning, Corning, NY, USA) without shaking. The media were carefully changed every other day without dislodging the spheroids.

For cells cultured in high-viscosity media, 3D spheroids were obtained from the Ready-to-use 3D Human Hepatic Stellate Cell Monoculture Spheroids (ScienCell Research Laboratories, Carlsbad, CA, USA), following the manufacturer’s instructions. One day after seeding, 60–70% of the top layer of the medium was changed using a pipette by hand to remove the residual DMSO. After the first medium change, no additional medium changes were necessary until treatment.

Cryopreserved, immortalized LX-2 human hepatic stellate cells were obtained from Millipore (Burlington, MA, USA). To culture stellate cells, a standard procedure was followed outlined in the manufacturer’s instructions for thawing, splitting and freezing of the cell line. LX-2 cells were cultured in DMEM high glucose with 2% FBS, 1× Pen/Strep, 1× Glutamine media in all experiments unless otherwise specified in the method.

Recombinant Human TGFβ protein was bought from Novus Biologicals (Centennial, CO, USA). An optimal TGFβ dose for treatment was tittered and selected for treating LX-2 cells with concentrations of 1, 5, 10, 20, 50 ng/mL, respectively, for 24 h (Figure S2). For the TGFβ treatment, 5 ng/mL of reconstituted TGFβ was added to the media. Cells were treated for 24 h before collection. Specifically for the 3D spheroids in the high-viscosity media, prepare double-concentrated TGFβ in the media and change only 50% of the top layer of the media to keep the spheroids intact.

### 4.2. Immunofluorescence Staining

HSC spheroids were fixed in neutral buffered formalin (Fisher Scientific, Newark, DE, USA), washed three times in PBS and blocked using goat serum diluted in PBS containing Triton X100. Spheroids were then labeled using antibodies against pan-collagen (Invitrogen, Rockford, IL, USA, Cat. PA1-36058) and ACAT2 (Sigma-Aldrich, St. Louis, MO, US, Cat. A2547). Secondary antibodies were then used to fluorescently indicate primary labeling, and DAPI was added as a nuclear counterstain. The labeled spheroids were imaged at 10× with 10 μm z-steps using a Molecular Devices ImageXpress Micro Confocal High-Content (Invitrogen, Fredrick, MD, USA) imaging platform.

### 4.3. RNA Isolation and RT-qPCR

HSCs were collected after treatment. For 3D spheroids, cells were collected via centrifugation at 10,000 rpm for 5 min at 4 °C. Total RNA was isolated by the MagMAX™-96 Total RNA Isolation Kit (Invitrogen, Fredrick, MD, USA) using the Kingfisher Duo Prime Purification System (Thermo Fisher Scientific, Pittsburg, PA, USA) with DNase treatment. An amount of 1 μg of isolated RNA was used for the reverse transcription using SuperScript III First-Strand Synthesis system (Invitrogen, Fredrick, MD, USA), following manufacturer’s instructions. Quantitative real-time RT-PCR was performed on a ViiA 7 Real-Time PCR System (Applied Biosystems Inc, Pittsburg, PA, USA) under the following conditions: 2 min 30 s at 95 °C for enzyme activation, 40 cycles of 15 s at 95 °C and 1 min at 60 °C. The melting curves were generated, and the analysis was conducted using the 2^−ΔΔCT^ method. The full primer sequences used are provided in Table S2.

### 4.4. RNA Sequencing and Bioinformatics Analysis

For bulk RNA-Seq, the strand-specific sequencing libraries were constructed using the TruSeq Stranded Total RNA Prep kit (Illumina, San Diego, CA, USA). Illumina HiSeq 3000 paired-end sequencing platform was used for sequencing at NHLBI DNA Sequencing and Genomics Core. The public database was downloaded from the Sequence Read Archive (SRA) database (https://www.ncbi.nlm.nih.gov/geo/, accession number: GSE85910, accessed on 8 March 2017; GSE134555, accessed on 26 August 2019; GSE142417, accessed on 20 October 2020). SRA files were unzipped using fastq-dump (sratoolkit, v3.1.1). Both homemade and public raw reads were trimmed by Cutadapt (v4.7). The raw reads were subjected to quality control by FastQC and alignment with human GENCODE v38 genome reference by HISAT2. FeatureCounts (subread/2.0, http://subread.sourceforge.net/, accessed on 20 October 2020) was then used to calculate the aligned reads, while differential expression genes (DEGs) were analyzed using the R package DESeq2/3.1.0 (https://bioconductor.org/packages/release/bioc/html/DESeq2.html, accessed on 20 October 2020). The drawing of overlapping and Venn diagrams was conducted with the online tool (https://bioinformatics.psb.ugent.be/webtools/Venn/, accessed on 20 October 2020). Pathway analysis was conducted by submitting the DEGs to the GO biological process (BP) using R package clusterProfiler/4.4.4 (https://bioconductor.org/packages/release/bioc/html/clusterProfiler.html, accessed on 20 October 2020). The top enriched pathways were visualized using the dot plot function.

Genes related to target lncRNAs in VSMC and stellate cells were determined computationally using the topological overlap matrix generated by the pyWGCNA package (pyWGCNA, https://doi.org/10.1093/bioinformatics/btad415, accessed on 20 October 2020). Default settings were used, except in the case of the soft thresholding power, which reflected the sample size (stellate cell = 8, VSMC = 6) according to recommendations put forward by the author of the original WGCNA package (WGCNA, https://bmcbioinformatics.biomedcentral.com/articles/10.1186/1471-2105-9-559, accessed on 20 October 2020). Stellate cell samples were first batch-corrected using the ComBat implementation in the InMoose python package (InMoose, https://github.com/epigenelabs/inmoose, accessed on 20 October 2020). The VSMC data used are publicly available under GEO accession GSE142417; stellate cell data are a combination of our own samples and data from accessions GSE219088, GSE210718 and GSE232640. The top 300 correlated genes were selected, with the enriched pathways analyzed using BioPlannet/).

### 4.5. Treatment of LX-2 Cells with CARMN Knockdown

For the CARMN GapmeR (Qiagen, Germantown, MD, USA) or ASO (Sigma-Aldrich, St. Louis, MO, USA) treatment, LX-2 cells were plated at 70% confluence in 24-well plates and incubated at 37 °C overnight. DharmaFect 1 reagent (Horizon Discovery, Cambridge, UK) was prepared to a final concentration of 1 μL per well. This transfection reagent was combined with 50 nM of CARMN or control GapmeR/ASO, and the media were changed to the transfection mix solution. After 6 h, the media were changed to regular LX-2 media with or without 5 ng/mL of TGFβ, and the cells were cultured for another 24 h. After a 24 h incubation, the media were removed, and cells were rinsed with 1× PBS solution. Cells could be used immediately for experiments or be snap-frozen using liquid nitrogen and stored at −80 °C. The knockdown tool sequences used are provided in Table S2. Antisense LNA GapmeR negative control was obtained from Qiagen. siRNA Universal Negative Control was obtained from Sigma.

### 4.6. Rapid Amplification of cDNA Ends (RACE)

The RACE assay to amplify the full-length transcript of CARMN was conducted using FirstChoice™ RLM-RACE Kit (Thermo Fisher Scientific, Pittsburg, PA, USA), following the manufacturer’s instructions. cDNA from LX-2 cells was used to conduct the RACE assay. The CARMN-specific primers used are provided in Table S2.

### 4.7. Cellular Fractionation

The cellular fractionation assay was conducted using NE-PER™ Nuclear and Cytoplasmic Extraction Reagents (Thermo Fisher Scientific, Pittsburg, PA, USA), following the manufacturer’s instructions. The input cells were LX-2 cells treated/untreated with 5 ng/mL TGFβ for 24 h. RNA was extracted from subcellular fractions. RT-qPCR was performed to determine the gene expression of CARMN and other lncRNAs (NEAT1 and DANCE) of the fractionated samples, setting the cytosolic expression level as 1. The primers used are provided in Table S2.

### 4.8. Western Blot

After cells were plated in 6-well plates (2 wells per treatment), cultured and treated, as described in Method 4.4, cells were lysed in 1% SDS lysis buffer containing phosphatase inhibitors (Sigma-Aldrich, St. Louis, MO, USA) and 100× Halt Protease Inhibitor Cocktail (Life Technology, Fredrick, MD, USA). The BCA method was used to quantify the protein lysates. Western blot was conduct by running the same amount of the lysate in SDS–PAGE, transferring proteins to PVDF membranes and then incubating the membrane with the primary antibodies (ACTA2: R&D Systems (Minneapolis, MN, USA), Cat. MAB1420; COL1A1: Cell Signaling Technology (Danvers, MA, USA), Cat. 39952; LOX: Novus (Centennial, CO, USA), Cat. NBP2-24877; PDGFRβ: Novus, Cat. AF385; TGFβ: Cell Signaling Technology, Cat. 41896; FN1: Novus, Cat. NBP2-22113, GAPDH, Cell Signaling Technology, Cat. 2118) at 4 °C overnight. The fluorescence conjugated secondary antibody (LI-COR, Lincoln, NE, USA) was incubated at room temperature the next day. The bound antibody was visualized using LI-COR quantitative fluorescence imaging system.

### 4.9. Collagen Staining

After 24 h incubation with 5 ng/mL TGFβ or solvent, cells were fixed and stained according to manufacturer’s instructions for staining of in vitro cell layers using Sirius Red/Fast Green Collagen Staining Kit (Chondrex, Woodinville, WA, USA). Cells were imaged using EVOS M5000 Imaging System (Thermo Fisher Scientific, Pittsburg, PA, USA) following staining.

### 4.10. Wound Healing Assay

Culture-Inserts 2 Well for self-insertion (Ibidi, Fitchburg, WI, USA) were placed into each well of a 12-well plate using sterilized forceps. The cells were plated and incubated overnight. The following day, cells were transfected with 50 nM CARMN or control GapmeRs. After 4 h, a Mitomycin C solution (Sigma-Aldrich, St. Louis, MO, USA) was added to each well to eliminate the contribution of cell proliferation to wound healing. After 2 h, the molds were carefully removed, and the media were replaced with 5 ng/mL TGFβ treatment. Wells were imaged at 0 and 17 h time points after TGFβ treatment to assess wound closure.

### 4.11. EdU Assay

For each condition, eight repeats were set in 96-well plates. Cells were plated and incubated overnight. They were transfected with 50 nM GapmeR siControl or siCARMN and incubated for 6 h. The media were changed to 5 ng/mL TGFβ treatment and incubated for 24 h before the EdU assay was carried out. The EdU assay was carried out according to the manufacturer’s instructions of Click-iT EdU Proliferation Assay for Microplates (Invitrogen, Fredrick, MD, USA). The wells were analyzed using the Synergy H4 Hybrid Reader (BioTeck, Santa Clara, CA, USA).

### 4.12. Adenovirus Production and LX-2 Infection

The overexpression construct of CARMN was generated via PCR amplification of the full-length sequence using the cDNA template from LX-2. The sequence was subsequently cloned to pAD/CMV/V5-DEST Gateway Vector (Invitrogen, Fredrick, MD, USA) according to the manufacturer’s protocols. The virus was prepared via 293A cell transfection and was purified via CsCl gradient centrifugation. The purified adenovirus was desalted with PD10 columns (GE Healthcare Life Sciences, Chicago, IL, USA) and tittered with the Adeno-X Rapid Titer Kit (Clontech, San Jose, CA, USA). LX-2 cells were infected with the purified adenovirus with MOI = 10 for 6 h. The media were changed, and the cells were treated with 5 ng/mL TGFβ for 24 h.

### 4.13. CRISPR Activation

The sgRNAs targeting human CARMN were designed using the online tool CRISPick from Broad Institute (https://portals.broadinstitute.org/gppx/crispick/public, accessed on 20 October 2020) with the following parameters: Reference Genome as Human GRCh38, Mechanism as CRISPRa, Enzyme as SpyoCas9 Hsu (2023) tracrRNA, CRISPick Quota as 10. Three non-overlapping sgRNAs were selected from the top ten candidates to be best represented, which were located 111 bp, 141 bp and 135 bp upstream of TSS of CARMN, respectively. The sgRNA was ligated into the sgRNA (MS2) cloning backbone (Addgene, 61424) using Golden-Gate reaction (SAM) after Golden-Gate annealing. The sgRNA vector was co-transfected with dCAS9-VP64-GFP (Addgene, 61422) and MS2-P65-HSF1_GFP (Addgene, 61422) with a ratio of 1:1:1 to the LX-2 cells using FuGENE HD Transfection Reagent (Promega, Madison, WI, USA), following the manufacturer’s instructions. The sgRNA seed sequences are provided in Table S2. The media were changed after 6 h, and the cells were treated with TGFβ using the same procedure as described above.

### 4.14. Statistics

For comparisons between two groups, a two-tailed, unpaired Student’s *t*-test was used. A *p*-value of more than 0.05 was considered non-significant (ns); a *p*-value between 0.01 and 0.05 was considered significant (*); a *p*-value between 0.001 and 0.01 was considered very significant (**); a *p*-value between 0.0001 and 0.001 was considered very extremely significant (***); a *p*-value of less than 0.0001 was considered extremely significant (****).

## Data Availability

The public database was downloaded from the Sequence Read Archive (SRA) database (https://www.ncbi.nlm.nih.gov/geo/, accession number: GSE85910, accessed on 8 March 2017; GSE134555, accessed on 26 August 2019; GSE142417, accessed on 20 October 2020). The RNA-Seq data of primary hepatic stellate cells (HSCs) and the LX-2 cell line treated with TGFβ were deposited in GEO with reviewer access (accession number: GSE253493, reviewer token: evmjiycytreffuv).

## Supplementary Materials

The following supporting information can be downloaded at: https://www.mdpi.com/article/10.3390/ncrna10030034/s1, Figure S1: Immunostaining of HSC 3D spheroids; Figure S2: TGFβ dose titers for in vitro treatment; Figure S3: Correlation pathways of three overlapped lncRNAs in smooth muscle cells and stellate cells; Figure S4: Expression of HSC activation panel markers using anti-sense oligos (ASOs) to knock down CARMN with TGFβ treatment. Table S1: Differentially expressed genes filtered by overlapping HSC with four other cell types with treatment; Table S2: Nucleotide sequence list; Table S3: Donor information; Table S4: Correlation gene list for CARMN, Linc01711 and RP11-154H23.3.

## References

[1] Powell E.E., Wong V.W., Rinella M. (2021). Non-alcoholic fatty liver disease. Lancet.

[2] Bellentani S., Scaglioni F., Marino M., Bedogni G. (2010). Epidemiology of non-alcoholic fatty liver disease. Dig. Dis..

[3] Machado M.V., Diehl A.M. (2016). Pathogenesis of Nonalcoholic Steatohepatitis. Gastroenterology.

[4] Friedman S.L., Neuschwander-Tetri B.A., Rinella M., Sanyal A.J. (2018). Mechanisms of NAFLD development and therapeutic strategies. Nat. Med..

[5] Tacke F., Puengel T., Loomba R., Friedman S.L. (2023). An integrated view of anti-inflammatory and antifibrotic targets for the treatment of NASH. J. Hepatol..

[6] Attia S.L., Softic S., Mouzaki M. (2021). Evolving Role for Pharmacotherapy in NAFLD/NASH. Clin. Transl. Sci..

[7] Deng K.Q., Huang X., Lei F., Zhang X.J., Zhang P., She Z.G., Cai J., Ji Y.X., Li H. (2022). Role of hepatic lipid species in the progression of nonalcoholic fatty liver disease. Am. J. Physiol. Cell Physiol..

[8] Friedman S.L., Roll F.J. (1987). Isolation and culture of hepatic lipocytes, Kupffer cells, and sinusoidal endothelial cells by density gradient centrifugation with Stractan. Anal. Biochem..

[9] Hautekeete M.L., Geerts A. (1997). The hepatic stellate (Ito) cell: Its role in human liver disease. Virchows Arch..

[10] Ramadori G., Saile B. (2002). Mesenchymal cells in the liver--one cell type or two?. Liver.

[11] Mann D.A., Marra F. (2010). Fibrogenic signalling in hepatic stellate cells. J. Hepatol..

[12] Gandhi C.R. (2017). Hepatic stellate cell activation and pro-fibrogenic signals. J. Hepatol..

[13] Tsuchida T., Friedman S.L. (2017). Mechanisms of hepatic stellate cell activation. Nat. Rev. Gastroenterol. Hepatol..

[14] Zisser A., Ipsen D.H., Tveden-Nyborg P. (2021). Hepatic Stellate Cell Activation and Inactivation in NASH-Fibrosis-Roles as Putative Treatment Targets?. Biomedicines.

[15] Seifuddin F., Singh K., Suresh A., Judy J.T., Chen Y.C., Chaitankar V., Tunc I., Ruan X., Li P., Chen Y. (2020). lncRNAKB, a knowledgebase of tissue-specific functional annotation and trait association of long noncoding RNA. Sci. Data.

[16] Iyer M.K., Niknafs Y.S., Malik R., Singhal U., Sahu A., Hosono Y., Barrette T.R., Prensner J.R., Evans J.R., Zhao S. (2015). The landscape of long noncoding RNAs in the human transcriptome. Nat. Genet..

[17] Statello L., Guo C.J., Chen L.L., Huarte M. (2021). Gene regulation by long non-coding RNAs and its biological functions. Nat. Rev. Mol. Cell Biol..

[18] Takahashi K., Yan I., Haga H., Patel T. (2014). Long noncoding RNA in liver diseases. Hepatology.

[19] Sallam T., Jones M.C., Gilliland T., Zhang L., Wu X., Eskin A., Sandhu J., Casero D., Vallim T.Q., Hong C. (2016). Feedback modulation of cholesterol metabolism by the lipid-responsive non-coding RNA LeXis. Nature.

[20] Batista P.J., Chang H.Y. (2013). Long noncoding RNAs: Cellular address codes in development and disease. Cell.

[21] Ruan X., Li P., Chen Y., Shi Y., Pirooznia M., Seifuddin F., Suemizu H., Ohnishi Y., Yoneda N., Nishiwaki M. (2020). In vivo functional analysis of non-conserved human lncRNAs associated with cardiometabolic traits. Nat. Commun..

[22] Ruan X., Li P., Ma Y., Jiang C.F., Chen Y., Shi Y., Gupta N., Seifuddin F., Pirooznia M., Ohnishi Y. (2021). Identification of human long noncoding RNAs associated with nonalcoholic fatty liver disease and metabolic homeostasis. J. Clin. Investig..

[23] Necsulea A., Soumillon M., Warnefors M., Liechti A., Daish T., Zeller U., Baker J.C., Grutzner F., Kaessmann H. (2014). The evolution of lncRNA repertoires and expression patterns in tetrapods. Nature.

[24] Diederichs S. (2014). The four dimensions of noncoding RNA conservation. Trends Genet..

[25] Sato M., Suzuki S., Senoo H. (2003). Hepatic stellate cells: Unique characteristics in cell biology and phenotype. Cell Struct. Funct..

[26] Meurer S.K., Weiskirchen S., Tag C.G., Weiskirchen R. (2023). Isolation, Purification, and Culture of Primary Murine Hepatic Stellate Cells: An Update. Methods Mol. Biol..

[27] Weiskirchen S., Tag C.G., Sauer-Lehnen S., Tacke F., Weiskirchen R. (2017). Isolation and Culture of Primary Murine Hepatic Stellate Cells. Methods Mol. Biol..

[28] Dang T.M., Le V.T., Do H.Q., Nguyen V.T., Holterman A.X.L., Dang L.T.T., Phan N.C.L., Pham P.V., Hoang S.N., Le L.T. (2021). Optimization of the isolation procedure and culturing conditions for hepatic stellate cells obtained from mouse. Biosci. Rep..

[29] Gressner A.M., Weiskirchen R., Breitkopf K., Dooley S. (2002). Roles of TGF-beta in hepatic fibrosis. Front. Biosci..

[30] Rockey D.C., Du Q., Weymouth N.D., Shi Z. (2019). Smooth Muscle alpha-Actin Deficiency Leads to Decreased Liver Fibrosis via Impaired Cytoskeletal Signaling in Hepatic Stellate Cells. Am. J. Pathol..

[31] Liu S.B., Ikenaga N., Peng Z.W., Sverdlov D.Y., Greenstein A., Smith V., Schuppan D., Popov Y. (2016). Lysyl oxidase activity contributes to collagen stabilization during liver fibrosis progression and limits spontaneous fibrosis reversal in mice. FASEB J..

[32] Manabe R., Oh-e N., Sekiguchi K. (1999). Alternatively spliced EDA segment regulates fibronectin-dependent cell cycle progression and mitogenic signal transduction. J. Biol. Chem..

[33] Lambrecht J., Verhulst S., Mannaerts I., Sowa J.P., Best J., Canbay A., Reynaert H., van Grunsven L.A. (2019). A PDGFRbeta-based score predicts significant liver fibrosis in patients with chronic alcohol abuse, NAFLD and viral liver disease. EBioMedicine.

[34] Sufletel R.T., Melincovici C.S., Gheban B.A., Toader Z., Mihu C.M. (2020). Hepatic stellate cells—From past till present: Morphology, human markers, human cell lines, behavior in normal and liver pathology. Rom. J. Morphol. Embryol..

[35] Sohara N., Znoyko I., Levy M.T., Trojanowska M., Reuben A. (2002). Reversal of activation of human myofibroblast-like cells by culture on a basement membrane-like substrate. J. Hepatol..

[36] Bennett M.R., Sinha S., Owens G.K. (2016). Vascular Smooth Muscle Cells in Atherosclerosis. Circ. Res..

[37] Doran A.C., Meller N., McNamara C.A. (2008). Role of smooth muscle cells in the initiation and early progression of atherosclerosis. Arterioscler. Thromb. Vasc. Biol..

[38] Chen P.Y., Qin L., Li G., Wang Z., Dahlman J.E., Malagon-Lopez J., Gujja S., Cilfone N.A., Kauffman K.J., Sun L. (2019). Endothelial TGF-beta signalling drives vascular inflammation and atherosclerosis. Nat. Metab..

[39] Low E.L., Baker A.H., Bradshaw A.C. (2019). TGFbeta, smooth muscle cells and coronary artery disease: A review. Cell Signal.

[40] Jaffe M., Sesti C., Washington I.M., Du L., Dronadula N., Chin M.T., Stolz D.B., Davis E.C., Dichek D.A. (2012). Transforming growth factor-beta signaling in myogenic cells regulates vascular morphogenesis, differentiation, and matrix synthesis. Arterioscler. Thromb. Vasc. Biol..

[41] Geerts A. (2004). On the origin of stellate cells: Mesodermal, endodermal or neuro-ectodermal?. J. Hepatol..

[42] Wirz W., Antoine M., Tag C.G., Gressner A.M., Korff T., Hellerbrand C., Kiefer P. (2008). Hepatic stellate cells display a functional vascular smooth muscle cell phenotype in a three-dimensional co-culture model with endothelial cells. Differentiation.

[43] Bao L., Chu Y., Kang H. (2023). SNAI1-activated long non-coding RNA LINC01711 promotes hepatic fibrosis cell proliferation and migration by regulating XYLT1. Genomics.

[44] Ilieva M., Miller H.E., Agarwal A., Paulus G.K., Madsen J.H., Bishop A.J.R., Kauppinen S., Uchida S. (2022). FibroDB: Expression Analysis of Protein-Coding and Long Non-Coding RNA Genes in Fibrosis. Noncoding RNA.

[45] Ounzain S., Micheletti R., Arnan C., Plaisance I., Cecchi D., Schroen B., Reverter F., Alexanian M., Gonzales C., Ng S.Y. (2015). CARMEN, a human super enhancer-associated long noncoding RNA controlling cardiac specification, differentiation and homeostasis. J. Mol. Cell Cardiol..

[46] Dong K., Shen J., He X., Hu G., Wang L., Osman I., Bunting K.M., Dixon-Melvin R., Zheng Z., Xin H. (2021). CARMN Is an Evolutionarily Conserved Smooth Muscle Cell-Specific LncRNA That Maintains Contractile Phenotype by Binding Myocardin. Circulation.

[47] Ni H., Haemmig S., Deng Y., Chen J., Simion V., Yang D., Sukhova G., Shvartz E., Wara A., Cheng H.S. (2021). A Smooth Muscle Cell-Enriched Long Noncoding RNA Regulates Cell Plasticity and Atherosclerosis by Interacting with Serum Response Factor. Arterioscler. Thromb. Vasc. Biol..

[48] Flomerfelt F.A., Gress R.E. (2016). Analysis of Cell Proliferation and Homeostasis Using EdU Labeling. Methods Mol. Biol..

[49] Chen C.K., Blanco M., Jackson C., Aznauryan E., Ollikainen N., Surka C., Chow A., Cerase A., McDonel P., Guttman M. (2016). Xist recruits the X chromosome to the nuclear lamina to enable chromosome-wide silencing. Science.

