# Supplementary material for: An Integrative Transcriptome Subtraction Strategy to Identify Human lncRNAs That Specifically Play a Role in Activation of Human Hepatic Stellate Cells
Source: Noncoding RNA. 2024 Jun 6;10(3):34. doi: 10.3390/ncrna10030034 (PMC11206700; doi:10.3390/ncrna10030034)
Supplement: Supplementary file 1 [file ncrna-10-00034-s001.zip › Supplementary Figures.pdf]

Supplementary Figures

Figure S1. Maximum intensity projections and quantification of pan-collagen (a) and α-SMA (b) labeling in individual spheroids. Spheroids after two weeks of preculture and 24-hours of treatment. The vehicle Control (Upper) shows visibly less signals than the spheroids that were stimulated with 10 ng/mL TGFβ (Lower). DAPI (blue) contrast was stretched to accommodate visualization of all nuclei. Data represent mean ± SEM of pan-collagen/α-SMA integrated density normalized to spheroid volume. Statistical analysis between two groups is conducted by 2-tailed, unpaired Student's *t* test (n=6).

p-value between 0.01 and 0.05 was considered significant (*); p-value between 0.0001 and 0.001 was considered very extremely significant (***).

Figure S2. Different doses for TGFβ in vitro treatment in LX2 cells. LX2 cells were treated with different doses of TGFb for 24h. The induction fold change of three stellate cell activation target genes, ACTA2, COL1A1, and LOX, after treatment were determined by qPCR.

Figure S3. Correlation pathways of three overlapped lncRNAs in stellate cells (a, CARMN; c, Linc01711; e, RP11-154H23.3) and VSMC (b, CARMN; d, Linc01711; f, RP11-154H23.3). Top 300 correlated genes were selected, with enriched pathways analyzed using BioPlanet.

Figure S4. Expression of HSC activation panel makers using Anti-sense Oligos (ASOs) to knock down CARMN with TGFβ treatment (n=3). (a) Result from the first ASO, ASO CARMN-1; (b) Result from the second ASO, ASO CARMN-2. The knockdown efficiency is shown side-by-side. The result is a representative of three repeated experiments. Data is shown as the mean ± SEM. Statistic analysis between two groups are conducted by 2-tailed, unpaired Student's *t* test.

p-value between 0.001 and 0.01 was considered very significant (**); p-value between 0.0001 and 0.001 was considered very extremely significant (***); p-value of less than 0.0001 was considered extremely significant (****).

Figure S1

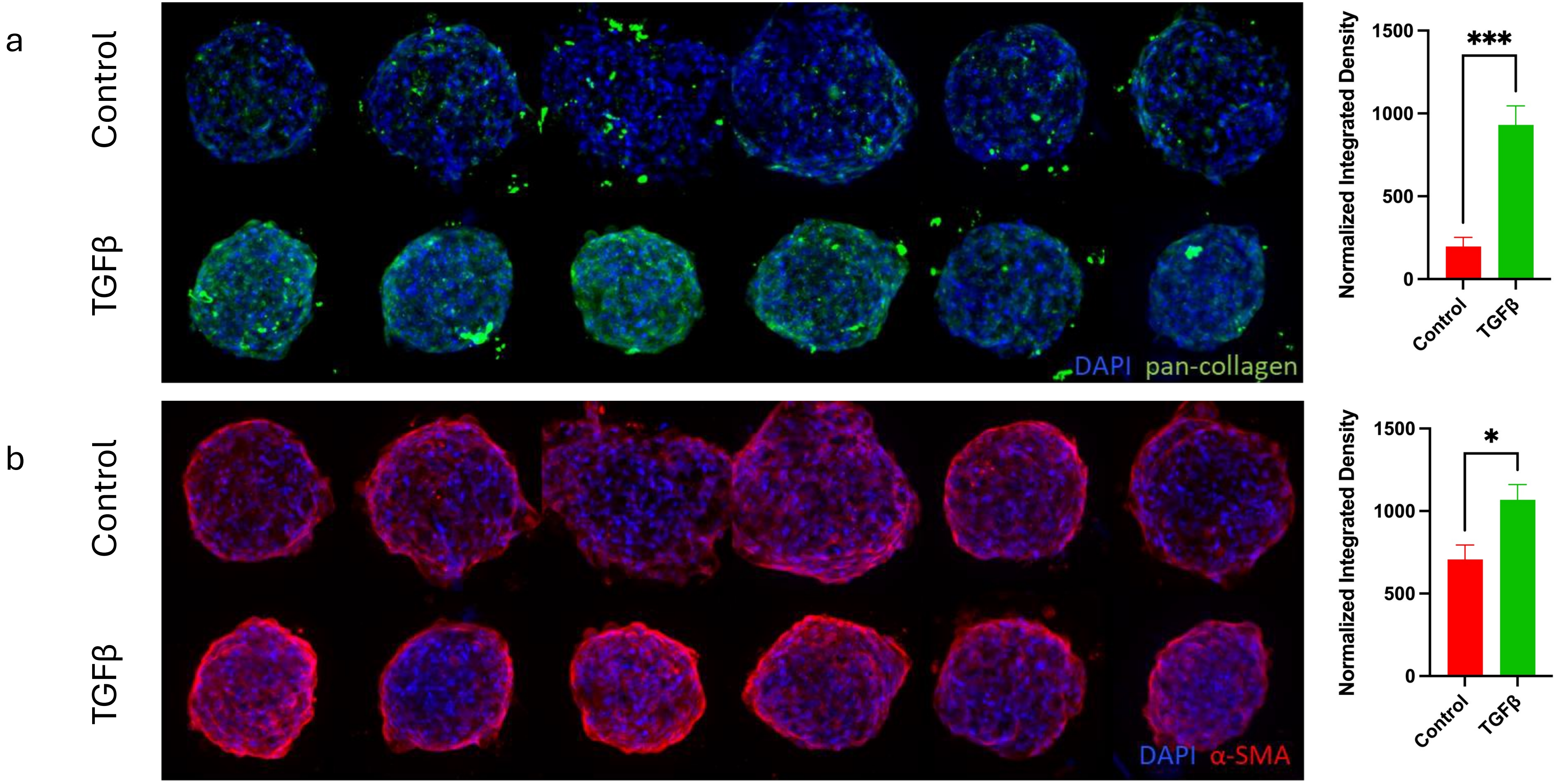
a
Control
TGFβ
DAPI pan-collagen
Normalized Integrated Density
1500
1000
500
0
***
Control
TGFβ
b
Control
TGFβ
DAPI α-SMA
Normalized Integrated Density
1500
1000
500
0
*
Control
TGFβ

Figure S2

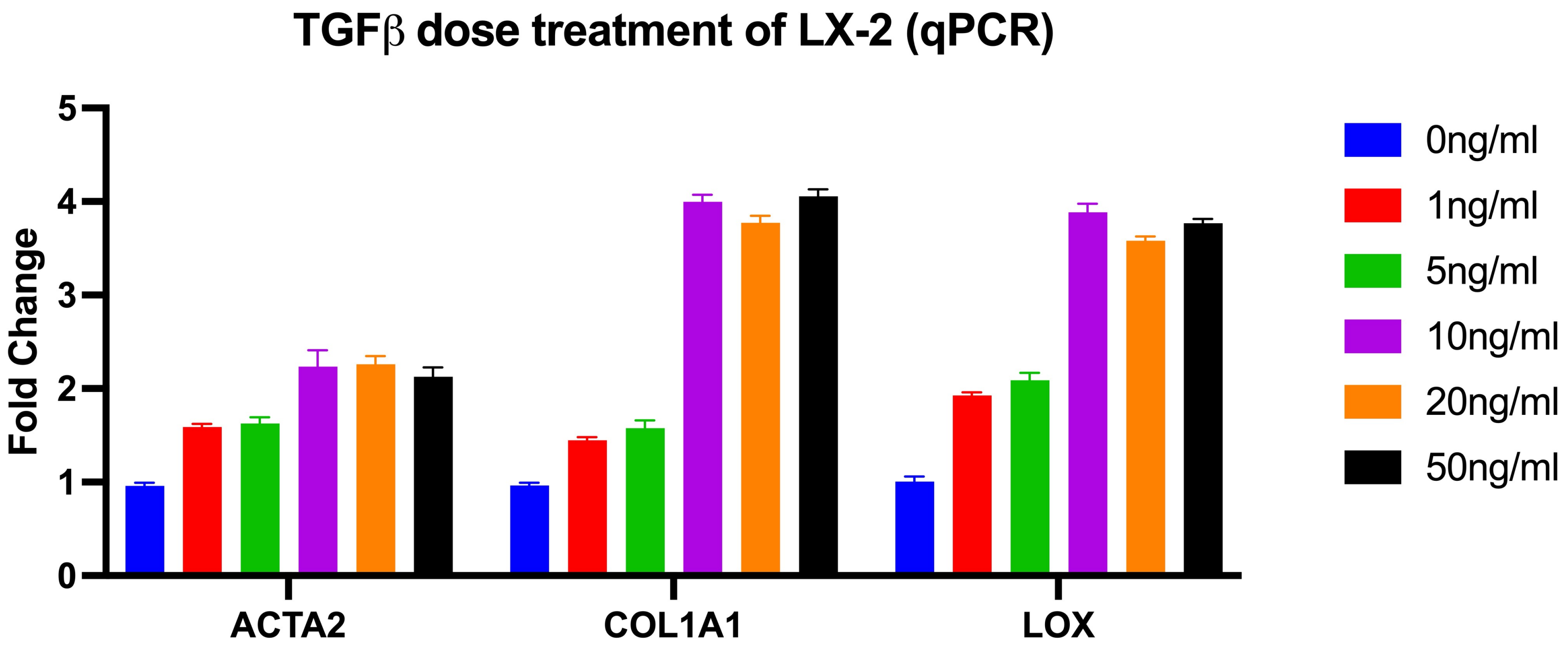
TGFβ dose treatment of LX-2 (qPCR)
Fold Change
0
1
2
3
4
5
ACTA2
COL1A1
LOX
0ng/ml
1ng/ml
5ng/ml
10ng/ml
20ng/ml
50ng/ml

Figure S3

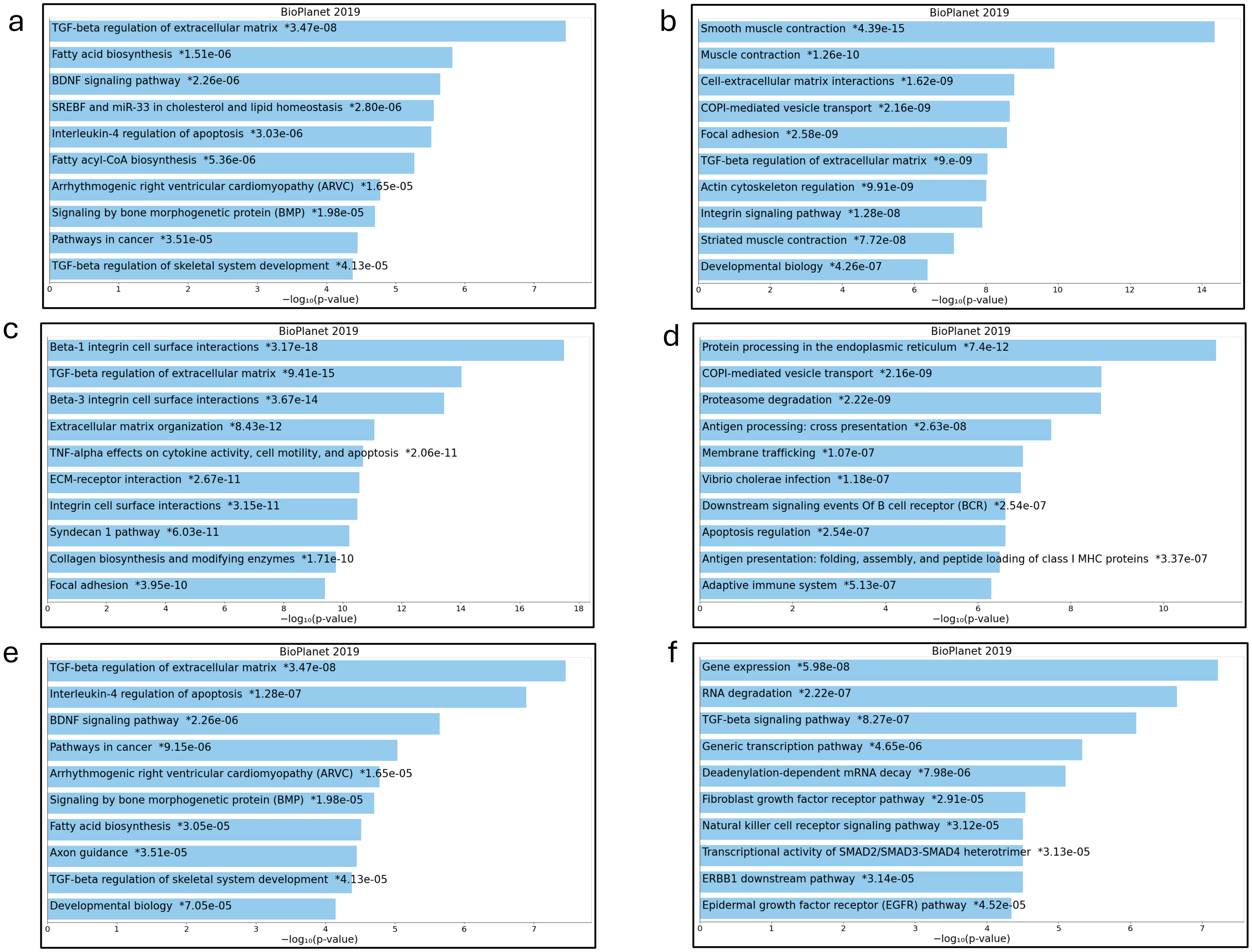
a
BioPlanet 2019
TGF-beta regulation of extracellular matrix *3.47e-08
Fatty acid biosynthesis *1.51e-06
BDNF signaling pathway *2.26e-06
SREBF and miR-33 in cholesterol and lipid homeostasis *2.80e-06
Interleukin-4 regulation of apoptosis *3.03e-06
Fatty acyl-CoA biosynthesis *5.36e-06
Arrhythmogenic right ventricular cardiomyopathy (ARVC) *1.65e-05
Signaling by bone morphogenetic protein (BMP) *1.98e-05
Pathways in cancer *3.51e-05
TGF-beta regulation of skeletal system development *4.13e-05
0 1 2 3 4 5 6 7
−log10(p-value)
b
BioPlanet 2019
Smooth muscle contraction *4.39e-15
Muscle contraction *1.26e-10
Cell-extracellular matrix interactions *1.62e-09
COPI-mediated vesicle transport *2.16e-09
Focal adhesion *2.58e-09
TGF-beta regulation of extracellular matrix *9.e-09
Actin cytoskeleton regulation *9.91e-09
Integrin signaling pathway *1.28e-08
Striated muscle contraction *7.72e-08
Developmental biology *4.26e-07
0 2 4 6 8 10 12 14
−log10(p-value)
c
BioPlanet 2019
Beta-1 integrin cell surface interactions *3.17e-18
TGF-beta regulation of extracellular matrix *9.41e-15
Beta-3 integrin cell surface interactions *3.67e-14
Extracellular matrix organization *8.43e-12
TNF-alpha effects on cytokine activity, cell motility, and apoptosis *2.06e-11
ECM-receptor interaction *2.67e-11
Integrin cell surface interactions *3.15e-11
Syndecan 1 pathway *6.03e-11
Collagen biosynthesis and modifying enzymes *1.71e-10
Focal adhesion *3.95e-10
0 2 4 6 8 10 12 14 16 18
−log10(p-value)
d
BioPlanet 2019
Protein processing in the endoplasmic reticulum *7.4e-12
COPI-mediated vesicle transport *2.16e-09
Proteasome degradation *2.22e-09
Antigen processing: cross presentation *2.63e-08
Membrane trafficking *1.07e-07
Vibrio cholerae infection *1.18e-07
Downstream signaling events Of B cell receptor (BCR) *2.54e-07
Apoptosis regulation *2.54e-07
Antigen presentation: folding, assembly, and peptide loading of class I MHC proteins *3.37e-07
Adaptive immune system *5.13e-07
0 2 4 6 8 10
−log10(p-value)
e
BioPlanet 2019
TGF-beta regulation of extracellular matrix *3.47e-08
Interleukin-4 regulation of apoptosis *1.28e-07
BDNF signaling pathway *2.26e-06
Pathways in cancer *9.15e-06
Arrhythmogenic right ventricular cardiomyopathy (ARVC) *1.65e-05
Signaling by bone morphogenetic protein (BMP) *1.98e-05
Fatty acid biosynthesis *3.05e-05
Axon guidance *3.51e-05
TGF-beta regulation of skeletal system development *4.13e-05
Developmental biology *7.05e-05
0 1 2 3 4 5 6 7
−log10(p-value)
f
BioPlanet 2019
Gene expression *5.98e-08
RNA degradation *2.22e-07
TGF-beta signaling pathway *8.27e-07
Generic transcription pathway *4.65e-06
Deadenylation-dependent mRNA decay *7.98e-06
Fibroblast growth factor receptor pathway *2.91e-05
Natural killer cell receptor signaling pathway *3.12e-05
Transcriptional activity of SMAD2/SMAD3-SMAD4 heterotrimer *3.13e-05
ERBB1 downstream pathway *3.14e-05
Epidermal growth factor receptor (EGFR) pathway *4.52e-05
0 1 2 3 4 5 6 7
−log10(p-value)

Figure S4

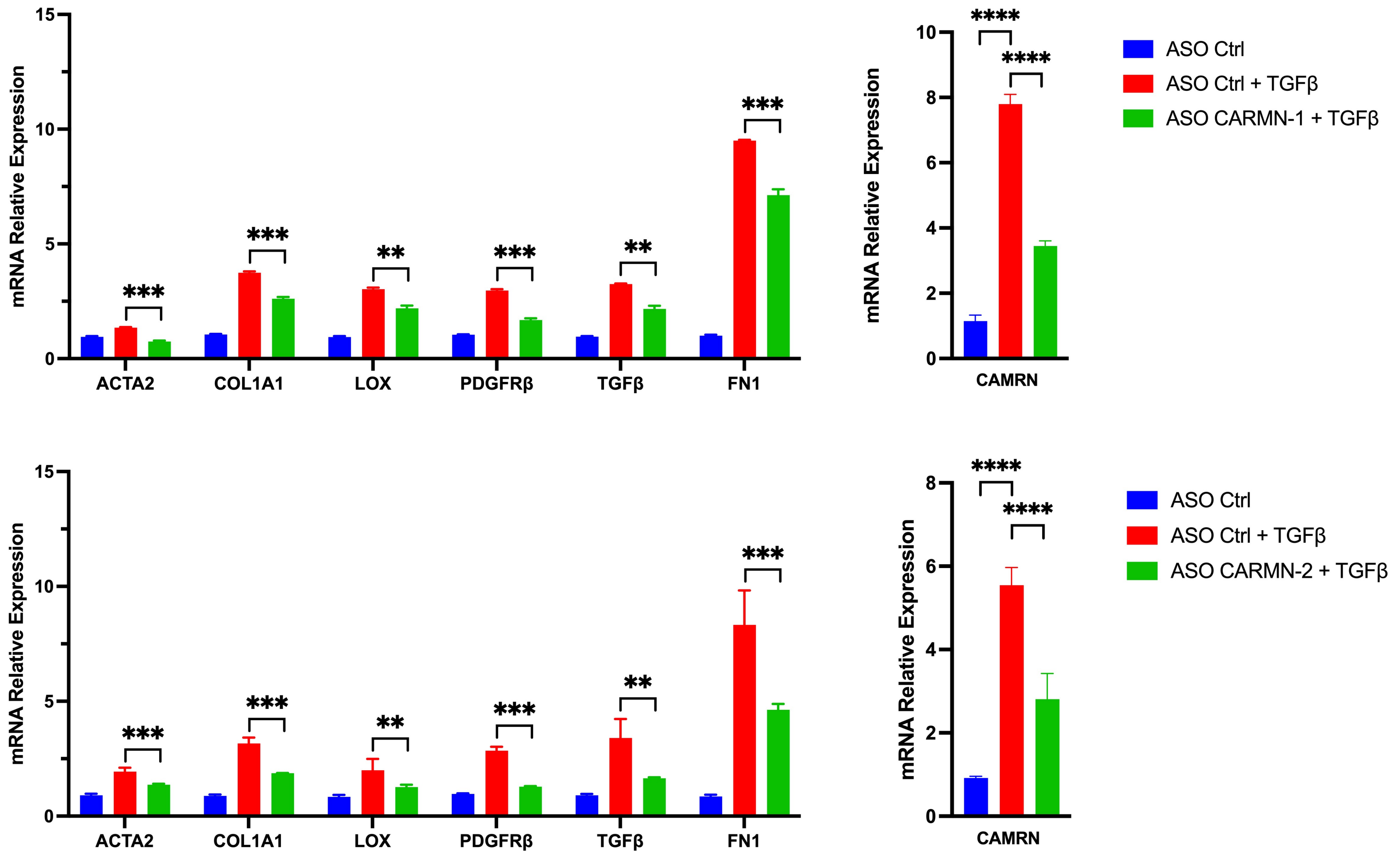

mRNA Relative Expression
15
10
5
0
ACTA2
COL1A1
LOX
PDGFRβ
TGFβ
FN1
***
**
CAMRN
****
ASO Ctrl
ASO Ctrl + TGFβ
ASO CARMN-1 + TGFβ
ASO CARMN-2 + TGFβ
